# Modification to Mirels scoring system location component improves fracture prediction for metastatic disease of the proximal femur

**DOI:** 10.1186/s12891-023-06182-7

**Published:** 2023-01-24

**Authors:** Richard L Amendola, Mark A Miller, Shannon M Kaupp, Richard J Cleary, Timothy A Damron, Kenneth A Mann

**Affiliations:** 1 Department of Orthopedic Surgery, SUNY Upstate Medical University, 750 East Adams Street, Syracuse, NY 13210 USA; 2 Division of Mathematics and Science, Babson College, 231 Forest St, Babson Park, MA 02457 USA

**Keywords:** Mirels scoring, Proximal femur, Pathologic fracture, Finite element, Decision curve analysis

## Abstract

**Background:**

Correctly identifying patients at risk of femoral fracture due to metastatic bone disease remains a clinical challenge. Mirels criteria remains the most widely referenced method with the advantage of being easily calculated but it suffers from poor specificity. The purpose of this study was to develop and evaluate a modified Mirels scoring system through scoring modification of the original Mirels location component within the proximal femur.

**Methods:**

Computational (finite element) experiments were performed to quantify strength reduction in the proximal femur caused by simulated lytic lesions at defined locations. Virtual spherical defects representing lytic lesions were placed at 32 defined locations based on axial (4 axial positions: neck, intertrochanteric, subtrochanteric or diaphyseal) and circumferential (8 circumferential: 45-degree intervals) positions. Finite element meshes were created, material property assignment was based on CT mineral density, and femoral head/greater trochanter loading consistent with stair ascent was applied. The strength of each femur with a simulated lesion divided by the strength of the intact femur was used to calculate the Location-Based Strength Fraction (LBSF). A modified Mirels location score was next defined for each of the 32 lesion locations with an assignment of 1 (LBSF > 75%), 2 (LBSF: 51–75%), and 3 (LBSF: 0–50%).

To test the new scoring system, data from 48 patients with metastatic disease to the femur, previously enrolled in a Musculoskeletal Tumor Society (MSTS) cross-sectional study was used. The lesion location was identified for each case based on axial and circumferential location from the CT images and assigned an original (2 or 3) and modified (1,2, or 3) Mirels location score. The total score for each was then calculated. Eight patients had a fracture of the femur and 40 did not over a 4-month follow-up period. Logistic regression and decision curve analysis were used to explore relationships between clinical outcome (Fracture/No Fracture) and the two Mirels scoring methods.

**Results:**

The location-based strength fraction (LBSF) was lowest for lesions in the subtrochanteric and diaphyseal regions on the lateral side of the femur; lesions in these regions would be at greatest risk of fracture. Neck lesions located at the anterior and antero-medial positions were at the lowest risk of fracture. When grouped, neck lesions had the highest LBSF (83%), followed by intertrochanteric (72%), with subtrochanteric (50%) and diaphyseal lesions (49%) having the lowest LBSF. There was a significant difference (p < 0.0001) in LBSF between each axial location, except subtrochanteric and diaphyseal which were not different from each other (*p* = 0.96).

The area under the receiver operator characteristic (ROC) curve using logistic regression was greatest for modified Mirels Score using site specific location of the lesion (Modified Mirels-ss, AUC = 0.950), followed by a modified Mirels Score using axial location of lesion (Modified Mirels-ax, AUC = 0.941). Both were an improvement over the original Mirels score (AUC = 0.853).

Decision curve analysis was used to quantify the relative risks of identifying patients that would fracture (TP, true positives) and those erroneously predicted to fracture (FP, false positives) for the original and modified Mirels scoring systems. The net benefit of the scoring system weighed the benefits (TP) and harms (FP) on the same scale. At a threshold probability of fracture of 10%, use of the modified Mirels scoring reduced the number of false positives by 17–20% compared to Mirels scoring.

**Conclusions:**

A modified Mirels scoring system, informed by detailed analysis of the influence of lesion location, improved the ability to predict impending pathological fractures of the proximal femur for patients with metastatic bone disease. Decision curve analysis is a useful tool to weigh costs and benefits concerning fracture risk and could be combined with other patient/clinical factors that contribute to clinical decision making.

**Supplementary Information:**

The online version contains supplementary material available at 10.1186/s12891-023-06182-7.

## Introduction

Metastatic bone disease (MBD) is associated with risk of pathologic fracture due to loss of bone strength [11, 17]. Although most MBD does not require orthopedic intervention, prophylactic stabilization of the femur is considered regularly to avoid the negative consequences of a complete fracture [4]. Correctly identifying patients at risk of fracturing is often difficult. Mirels [20] developed a widely referenced scoring system with four components: pain level, type of lesion, size, and location. While sensitivity of Mirels criteria appears to be relatively strong (71–100%), specificity is poor (13–50%) [10, 13, 23, 30]. The advantage of the Mirels system is that it is widely known and easily calculated. Hence, if a simple modification of Mirels scoring improves specificity, a more accurate means of fracture prediction would be widely available.

One puzzling aspect of Mirels scoring is that the anatomic location criteria neither individually predicts fracture risk or improves the accuracy when included in the total score [20]. For the proximal femur, Mirels location score assigns a higher (worse) score to pertrochanteric regions compared to lesions of the femoral diaphysis. However, for activities of daily living (ADL), experimental [27] and computational [15] analyses of the intact proximal femurs, strain magnitudes are often higher in the diaphyseal and subtrochanteric regions compared to femoral neck and intertrochanteric regions. Further, within a single region, such as the femoral neck, the lesion’s location dramatically affects femoral fracture strength [3]. We posit that a more granular approach to assigning location score within the femur could improve the Mirels overall predictive capabilities.

In this study, computational (finite element) experiments were performed to quantify strength reduction in the proximal femur due to presence of lytic lesions at defined locations. From this, a modified Mirels scoring system was proposed and applied to a clinical data set of patients with femoral MBD and known outcome (fracture or no fracture). Decision curve analysis was used to determine the net benefit of using a modified Mirels score compared to the original Mirels scoring method. We asked three research questions: (1) Does proximal femoral strength reduction differ depending upon the specific anatomic site of the lesion? (2) Can a modified scoring metric based upon more specific femoral lesion location, informed by the finite element experiments, improve clinical fracture prediction when compared to original Mirels scoring? (3) What is the relative net benefit of using a modified scoring system over original scoring?

## Methods

### Study design

**Fig. 1:**
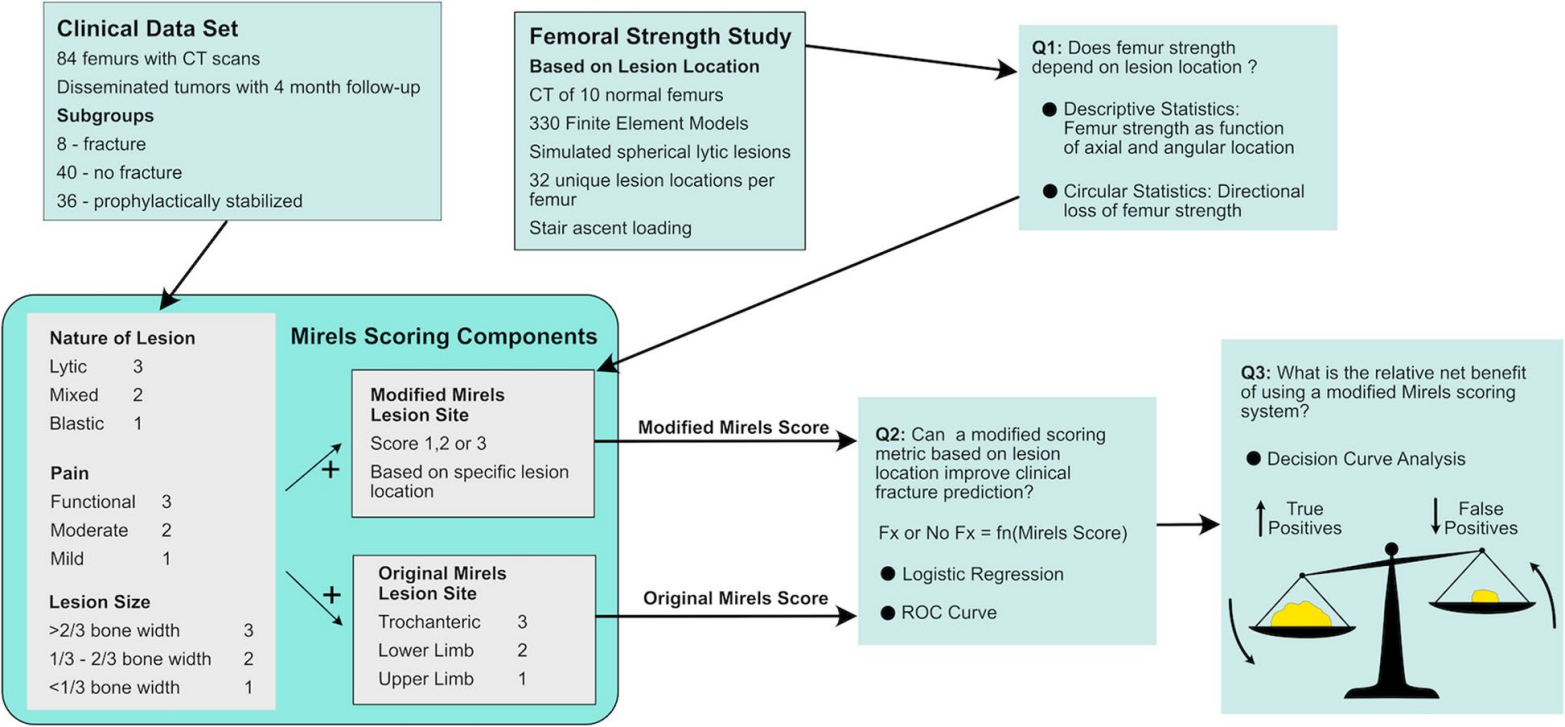
Workflow for the development of a modified Mirels scoring system. Legend: Finite element analysis of proximal femur strength using simulated stair ascent loading conditions was performed for models with 32 unique lesion locations per femur. Intact femurs without lesions served as controls. A modified lesion site score was then developed. Modified and original Mirels scores were determine for a clinical dataset of 84 patients with disseminated tumors of the proximal femur. Logistic regression and ROC curves were generated to determine if the modified Mirels score improved fracture prediction. Decision curve analysis was used to assess the relative net benefit of using the modified Mirels scoring over original Mirels scoring in terms of true and false positives

Computational simulations were first conducted to assess the strength of femurs with idealized lytic lesions placed at defined locations in the proximal femur. From this, a modified Mirels location score was determined based on axial and angular location of the lesions. The ability of the modified Mirels score to improve pathologic fracture prediction over original Mirels scoring was then evaluated for a clinical series of patients with disseminated tumors to the femur and known fracture status over a 4 month follow up period. Logistic regression and decision curve analysis were used to explore relationships between clinical outcome (fracture/no fracture) and the original and modified Mirels scoring methods. A schematic of the overall workflow for this project is illustrated in Fig. 1.

### Study subjects

The STROBE checklist for cross-sectional studies was used to guide reporting of clinical research. Seventy-two patients with disseminated tumors to the femur (total of 84 femurs) were previously enrolled in a Musculoskeletal Tumor Society (MSTS) study (July 2008 through April 2018). Eligibility criteria included patients evaluated for femoral bone involvement from disseminated metastatic carcinoma. Patients were included regardless of prognosis or expected treatment. Design of data collection was that of a diagnostic level III study. Some of these 84 femurs were analyzed for other purposes both as single institutional data or combined with multi-institutional data [5, 22, 23]. The most common primary cancers from the current cohort were breast, followed in rank order by multiple myeloma, lung, renal, and prostate cancer. Consenting individuals had quantitative Computed Tomography (CT) scans (Siemens Sensation 16, Erlangen, Germany or GE Medical Systems Lightspeed 16, Milwaukee Wisconsin) with a hydroxyapatite (HA) phantom prior to fixation or fracture. Three groups were identified: (1) fracture group (*n* = 8) sustained a proximal femoral fracture within four months of initial CT, (2) no fracture group (*n* = 40) did not sustain a fracture over 4 month follow up, and (3) prophylactically stabilized group (*n* = 36) surgically stabilized due to perceived high fracture risk as assessed by the orthopedic oncologist (TAD). The fracture and no fracture groups were used to compare fracture prediction of Mirels versus modified Mirels scoring in the current study. The relatively short 4 month follow-up window to assess fracture risk was chosen because fractures that occurred later (~ 1 year) would likely have progression of the metastatic lesions, but this would not be represented by the initial CT scan. However, the MSTS protocol did follow all patients to 12 months. Exclusion of the 36 stabilized femurs could bias the study through removal of cases because it is not known definitively if fracture would have occurred. A subset of patients (*n* = 12) had bilateral lesions that were scored as independent cases; these could potentially bias results as each of case is not truly independent.

### Demographics

Patient demographics were similar for the three groups in terms of age and weight (Table 1), although there was a much higher proportion of females in the fracture group. The original Mirels score (with each of its individual components) was recorded by TAD at enrollment in the study. Clinical course of action was not impacted by the current study, which was performed several years after completion of patient enrollment. Detailed information concerning primary caner, lesion location, original Mirels score, and time to fracture of the fracture group are listed in Table 2. Half of the fractures occurred within several days of the initial CT scan, prior to any scheduled surgery. Three cases fractured during the 4 month follow-up period. These cases had Mirels scores of 9, but pain scores of 1. One patient declined prophylactic stabilization. Details of all 48 cases is not included here, but a detailed pictorial review of cases with metastatic bone disease with prediction using Mirels scoring, CT-based structural rigidity analysis (CTRA), and finite element analysis (FEA) is described elsewhere [14].

**Table 1:** Patient demographics and original Mirels scoring

	***Fracture (Fx)***	***No Fracture (No Fx)***	***Fx vs No Fx*** t-test		***Stabilized***	***No Lesion***^a^
*N*	8	40		*N*	36	10
*Sex (F | M)*	7 | 1	26 | 14		*Sex (F | M)*	20 | 16	5 | 5
*Age (Years)*	64 [42–87]	62 [23–95]	*p* = 0.78	*Age (Years)*	63 [33–88]	66 [54–88]
*Weight (kg)*	83 [42–142]	78 [42–142]	*p* = 0.66	*Weight (kg)*	78 [46–122]	86 [46–115]
*BMI (kg/m*^*2*^*)*	28.5 [20.3–38.8]	28.5 [16.5–61.5]	*p* = 0.99	*BMI (kg/m*^*2*^*)*	26.5 [16.5–43.7]	30.7 [16.9 – 39.8]
*Original Mirels Score*	10.5 [9,10,11,12]	8.5 [6,7,8,9,10]	*p* = 0.0049	*Original Mirels Score*	10.6 [8,9,10,11,12]	NA

Seventy-two patients with disseminated tumors to the proximal femurs (*n* = 84) resulted in fracture, no fracture, or were prophylactically stabilized. An additional No Lesion (^a^) femur set was identified from the sample population and was used to create the finite element models with idealized spherical lesions. Mean and range shown. Two-sample t-tests indicate *p*-values for differences between fracture and no fracture groups

**Table 2:** Detailed demographics of the 8 fracture cases

	Primary Cancer	Axial Lesion Location	Angular Lesion Location	Original Mirels Score	Time to Fracture	Notes
1	Renal Cell	Diaphyseal	Anterior	11	0 days	Fracture at time of CT scan
2	Multiple Myeloma	Diaphyseal	Central	9	3 months	Fracture during follow-up (Mirels Pain 1)
3	Breast	Subtrochanteric	Posterior medial	12	4 days	Fracture prior to scheduled surgery
4	Multiple Myeloma	Diaphyseal	Posterior medial	9	2 months	Fracture during follow-up (Mirels Pain 1)
5	Anal	Femoral head	Medial	12	4 months	Declined prophylactic stabilization
6	Renal Cell	Subtrochanteric	Posterior medial	9	2 months	Fracture during follow-up (Mirels Pain 1)
7	Unknown	Subtrochanteric	Central	12	10 days	Intraoperative fracture
8	Breast	Diaphyseal	Posterior	10	0 days	Fracture at time of CT scan

Eight patients sustained fractures of the proximal femur during the 4 month follow-up period from time of initial CT scan

### Femoral strength reduction based on lesion location

Finite element models of femurs containing idealized spherical lytic lesions at specific locations were created and analyzed to determine the effect of lesion location on femur strength. It should be appreciated that a spherical lesion geometry is an idealized shape and each metastatic lesion has a unique morphology that could be explored in the future through principal component analysis of lesion shape for a large population of metastatic lesions. However, lesion shape is not currently an element of the Mirels scoring system and might be difficult to implement straightforwardly. The model simulations only consider lytic lesions; inclusion of mixed or blastic lesions in the models would likely change the predicted failure loads, but there is uncertainty concerning the mechanical properties of the different lesion types [2]. It should also be noted that the nature of the lesion (lytic, mixed, blastic) and lesion size are accounted for as part of the overall original and modified Mirels score (Fig. 1).

**Fig. 2:**
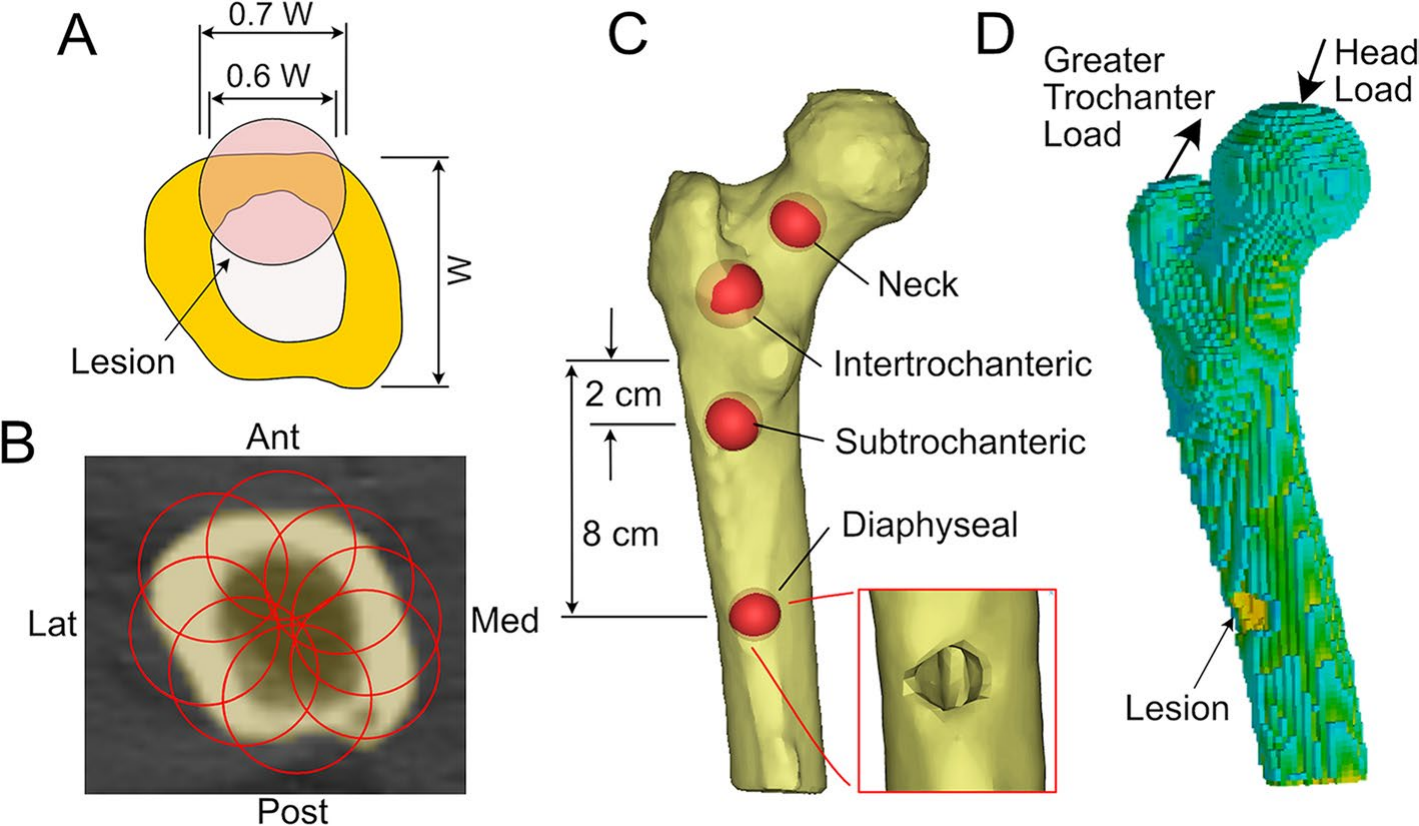
Femoral Strength based on lesion location. Legend: Spherical lesions (**A**) in the proximal femur were created using Boolean subtraction of the spherical lesion from the intact femur. Lesion size was scaled to the minor width of the bone (W) and had a width of 70% of the minor bone width. Eight lesions were individually placed (**B**) at each axial level. Lesions for the neck, intertrochanteric, subtrochanteric, and diaphyseal axial positions were included (**C**). Voxel based finite element models were created from the solid models with material assignment based on mineral equivalent density (**D**). A head and greater trochanter load was applied to represent stair ascent loading

CT-based solid models were generated (MIMICS 21, Materialise, Plymouth, MI) of the proximal half of ten normal femurs without lesions (Table 1) using a lower threshold of 100 mg/cc HA to identify bone. These femurs were chosen to achieve a sample distribution similar to the clinical fracture/no fracture groups in terms of age and weight. Virtual spherical defects representing lytic lesions were placed at defined locations in the femur and Boolean subtraction (femur minus sphere volume) was used to create unique solid models with a single lesion. The lesion diameter was 70% of bone minor section width, and the lesion pierced the periosteal bone surface such that the periosteal lesion width was 60% of the bone width (Fig. 2A). A large lesion size was chosen because femur strength is more sensitive to large lesions compared to small lesions. Eight anatomic-angular (eg, anterior, medial, lateral, posterior, located at 45-degree intervals, Fig. 2B)) and four axial (neck, intertrochanteric, subtrochanteric or diaphyseal, Fig. 2C) lesion locations were created per femur. The subtrochanteric and diaphyseal lesions were placed at distances 2 and 8 cm distal from center of the lesser trochanter, respectively. A total of 320 solid models with lesions (32 unique models/femur) were developed. Ten solid models without lesions were also included and served as the normal ‘controls’.

Voxel-based (2 × 2 x 2 mm) finite element meshes were created from the solid models with between 24,000 and 44,000 elements per model (Fig. 2D). Isotropic, but heterogeneous material property assignments were assigned to each voxel based on CT mineral density [21] and a Hoffman failure criteria with 0.667% compressive and 0.4% tensile yield strains for bone tissue was used [24]. Joint loads were applied to the femoral head and abductor loads to the greater trochanter to simulate stair ascent [10]. The distal end of the femur was fixed with a distal pot of graded modulus (100–1000 MPa) to prevent local stress concentrations in the bone. Models were analyzed using Marc/Mentat (MSC Software, Irvine, CA). The utility of the non-linear finite element modeling approach with stair climbing was previously validated using a clinical data set of patients with metastatic bone lesions [10]. A separate validation study with experimental (cadaver data) is included in the Appendix (Additional File 1). The strength of the femur with the lesion divided by strength of the intact femur was used to calculate the Location-Based Strength Fraction (LBSF) for each of the 320 femur/lesion location combinations. From the finite element modeling, the mean and standard deviation of the LBSF was determined at each axial-angular lesion location for the 10 femurs and a table of LBSF for the 32 lesion locations was created.

Modified mirels location scoring.

Using CT scans of the clinical data set, the lesion axial and angular location was identified for each fracture, no fracture, and stabilized case (Table 1). The modified Mirels score was calculated for each patient by replacing the original Mirels location score (MLS) with a modified Mirels location score (MMLS) using two different approaches. A MMLS-ss (*site-specific)* was based on both axial and angular lesion location information. The MMLS-ss was defined over a 1 to 3 range with an assignment of 1 (LBSF ≥ 75%), 2 (50 < LBSF < 75%), or 3 (LBSF ≤ 50%). A second MMLS-ax (*axial)* used the average value of all angular locations for each axial location. In this scenario, each of the four axial locations (diaphyseal, subtrochanteric, intertrochanteric, neck) was assigned a single MMLS-ax. If a lesion spanned two axial locations or there was more than one discrete lesion, the more distal was selected as the location. The rationale for this was that for two lesions of the same size, the more distal lesions resulted in lower LBSF, and thus would fracture first at the lower LBSF lesion. Diffuse or innumerable lesions were assigned the average LBSF for the proximal femur. For lesions that did not occupy any specific angular location, the average LBSF for the axial level was used. There were three cases with femoral head lesions that extended to the base of the femoral head; these were assigned corresponding LBSF values from the neck region. Lesion size was measured in the axial plane using the ratio of major diameter of the lesion to coaxial diameter of the bone.

### Statistical analysis

To determine if proximal femur strength reduction differed depending on anatomic lesion site, mean and standard deviation of the location-based strength fraction (LBSF) was determined for each axial location and anatomic-angular location and these were reported using polar coordinate plots. Because this data is reported on a circular scale (where 0 degrees = 360 degrees) rather than a linear scale, a circular statistics approach [35] was used to test for uniformity of loss of femur strength (100-LBSF) based on the angular location of the lesions. The concentration (r) and mean angle (θ) of loss of strength were determined for each individual femur at each axial position, and the vector mean for the 10 femurs was calculated. A nonparametric second order statistical analysis (mean of means) was performed to test for non-uniform (directional) loss of femur strength. Differences in LBSF based on axial position was evaluated using one-way ANOVA and Tukey–Kramer post-hoc test.

To determine if the modified scoring metrics could improve fracture prediction, logistic regression was performed for the 48 fracture/no fracture cases with fracture status as the dependent variable and the Mirels scoring schemes as independent variables. From these, the probability of fracture was estimated as a function of Mirels score. Receiver operator characteristic (ROC) curves were generated to explore the interaction between sensitivity and specificity of each of the Mirels scoring schemes in predicting femur fracture. JMP Pro 16 (SAS, Cary, NC) was used for statistical analyses.

### Decision curve analysis and net benefit

Interpreting the relative benefits and costs of a specific test is difficult when considering only sensitivity and specificity of each fracture prediction tool as shown by the ROC curves. Decision curve analysis (DCA) [32, 34] provides an approach to quantify the relative risks of identifying patients that will fracture (TP, true positives) and those erroneously predicted to fracture (FP, false positives). Net benefit (NB) analysis is a simple type of decision analysis with benefits (TP) and harms (FP) of the surgical procedure on the same scale so they can be compared directly. Net benefit using each scoring system was calculated from TP, FP, and the threshold probability of fracture (*p*_*t*_) for the subject population (N):

1 $$NB=\frac{TP}{N}-\frac{FP}{N}\left(\frac{{p}_{t}}{1-{p}_{t}}\right)$$

Key to this analysis is the interpretation of threshold probability (p_t_). As an example, a threshold probability of 20%, chosen by a surgeon, would weigh the benefit of finding and treating one true positive as the same as the cost of treating four false positives. As applied in Eq. 1, it is clear that a priori identification of a TP via Mirels scoring would add to the net benefit, while identifying a FP would be a cost that would subtract from the net benefit. The threshold probability provides the flexibility in defining how one wants to weigh TP and FPs for a particular scenario. Further, as threshold probability is an independent variable, comparisons between tests, or effectiveness of a single test can be determined over a full range of threshold probabilities.

The relative net benefit of using one fracture prediction scoring scheme over another can be assessed. The reduction in false positives for the modified Mirels scoring schemes relative to the original Mirels scoring scheme was calculated as a function of the threshold probability:

2 $$FP\;Reduction=\frac{{NB(p_t)}_{ModifiedMirels}-{NB(p_t)}_{Mirels}}{(p_t/\left(1-p_t\right))}x100forp_t>0$$

## Results

### Femoral strength reduction based on lesion location

**Fig. 3:**
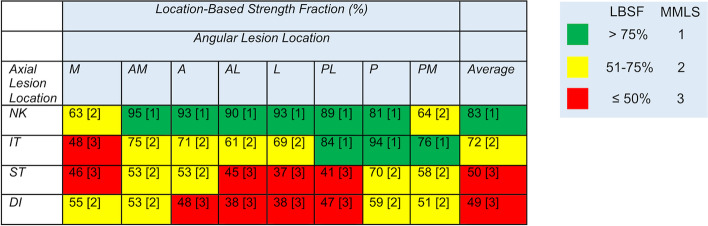
Location-Based Strength Fraction (LBSF) Results. Legend: A lookup table is used to identify the LBSF and the corresponding site-specific Modified Mirels Location Score (MMLS) shown in brackets [1–3]. For angular lesion location: M: medial, AM: antero-medial, A: anterior, AL: antero-lateral, L: lateral, PL: postero-lateral, P: posterior, PM: postero-medial. For axial lesion location: NK: neck, IT: intertrochanteric, ST: subtrochanteric, DI: diaphyseal

The location-based strength fraction (LBSF) was lowest for lesions in the subtrochanteric and diaphyseal regions on the lateral side (Fig. 3) indicating that femurs with lesions located in these regions would be most at risk of fracture. In contrast, neck lesions located at the anterior and antero-medial positions had the greatest LBSF and would be at the least risk of fracture. As a group, neck lesions had the highest LBSF, followed by intertrochanteric, with subtrochanteric and diaphyseal lesions having the lowest LBSF. There was a significant difference (p < 0.0001) in LBSF between each axial lesion location except subtrochanteric and diaphysis, which were not different (*p* = 0.96) from each other, tested using one-way ANOVA and Tukey–Kramer post-hoc test.

**Fig. 4:**
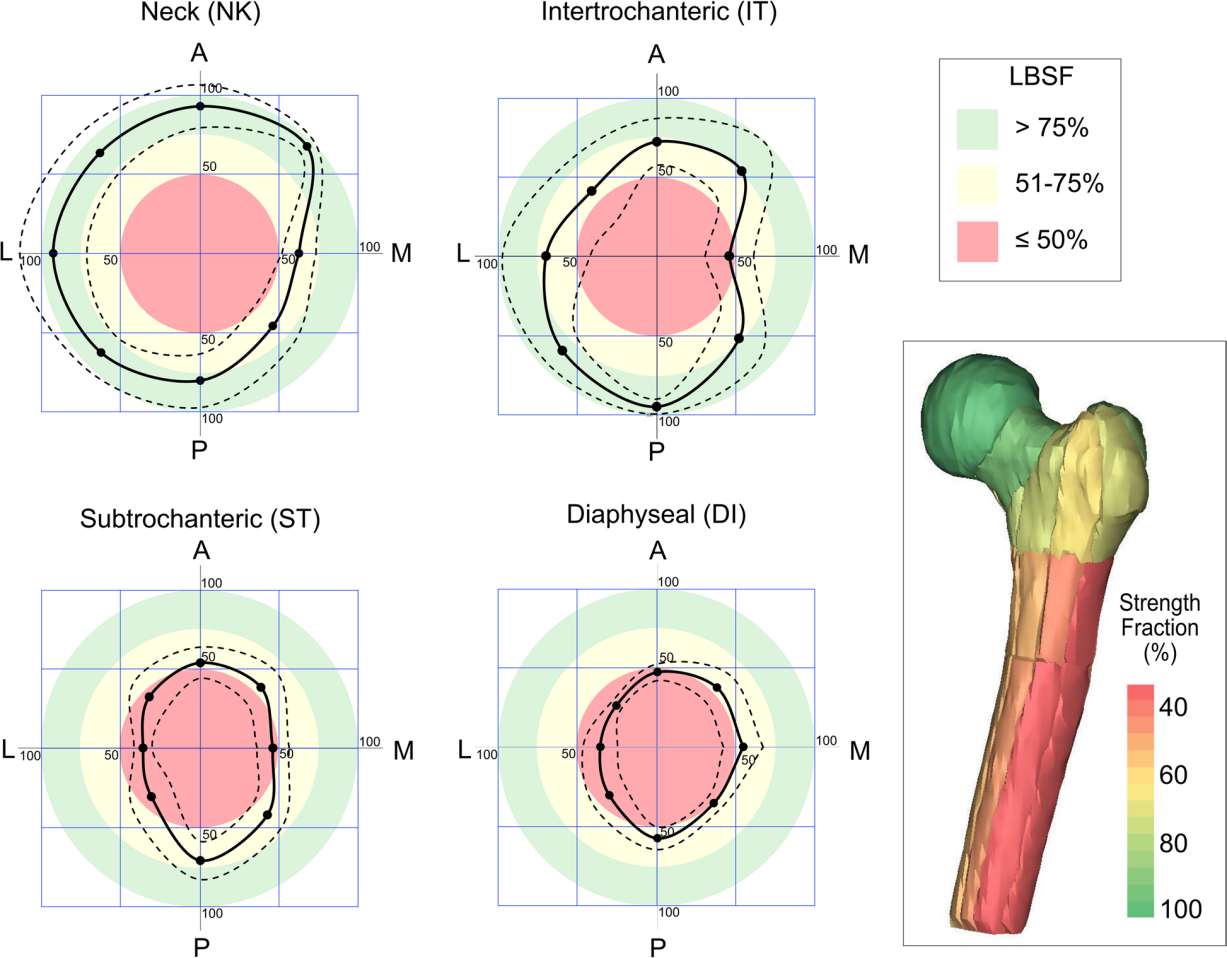
Polar plots of location-based strength fraction (LBSF) at the four axial levels for stair ascent loading. Legend: Mean (solid line) and standard deviation (dashed lines) for the 10 models at each axial/angular lesion location are shown. Anterior (A), Posterior (P), Medial (M), and Lateral (L) anatomic directions are indicated. Red, yellow, and green shaded regions correspond to the three LBSF ranges used in the modified Mirels location score. A heat map of the LBSF distribution on a proximal femur is shown to the right

For the intertrochanteric, subtrochanteric, and diaphyseal sections, there was some symmetry in the polar plots of LBSF (Fig. 4) with the greatest loss of strength found for lesions located in the lateral and medial locations compared to anterior and posterior. This is consistent with the large bending moment created by the femoral head and abductor load acting about the anterior–posterior axis during stair ascent. There was also some variance in LBSF measures at each axial/angular position due to geometry and material/density variations in the sample population of the 10 femur models. The data reported in Fig. 3 was taken from the mean results at each axial/angular position. Future work could explore the effect of uncertainty in the LBSF through statistical modeling.

**Fig. 5:**
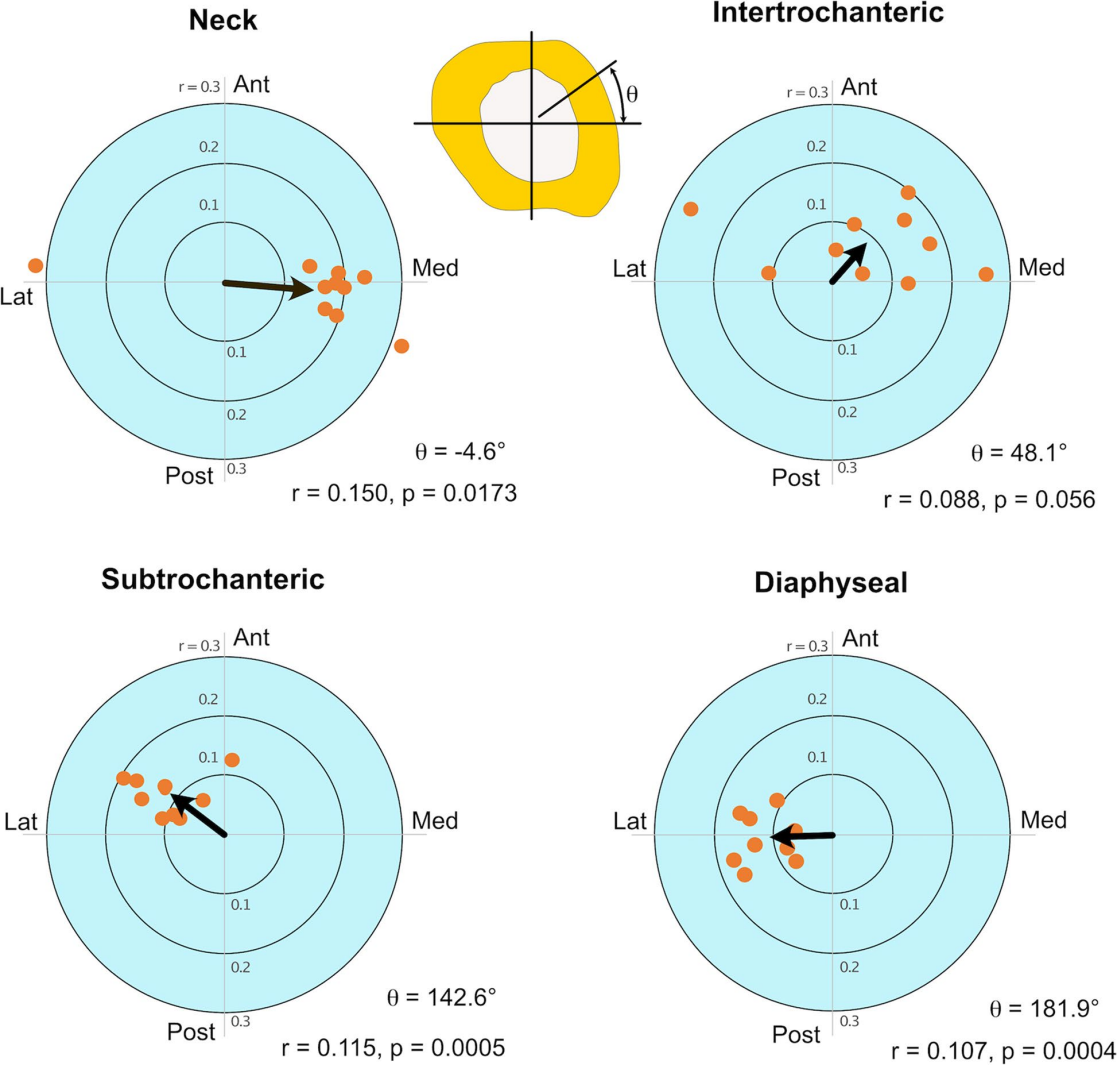
Circular statistics polar plots. Legend: Polar plots show directional concentration (r) and orientation (θ) for loss of femur strength (LOS) due to presence of lesions for the four axial positions. Data for each femur is shown as individual points and the vector mean (black arrow) represents the average of the 10 femurs

Using a circular statistics approach, loss of femur strength (100-LBSF) was reported in place of LBSF because the resulting concentration vector points in the direction of the weakest lesion location, rather than LBSF which would report the strongest direction. There was clustering of the direction of individual femur loss of strength at each axial position away from the origin of the plots (Fig. 5). The concentration (r) and mean angle (θ) of loss of femur strength showed that at the neck there was a directional loss of strength in the medial direction (*p* = 0.0173) as illustrated by the vector mean for the 10 femurs. The directional loss of strength shifted towards an anteromedial direction for intertrochanteric lesions, but the mean concentration was not statistically significant (*p* = 0.056). For the subtrochanteric and diaphyseal sections, the loss of strength shifted towards the antero-lateral (*p* = 0.0005) and lateral (*p* = 0.0004) angular directions, respectively.

### Modified mirels location scoring

**Fig. 6:**
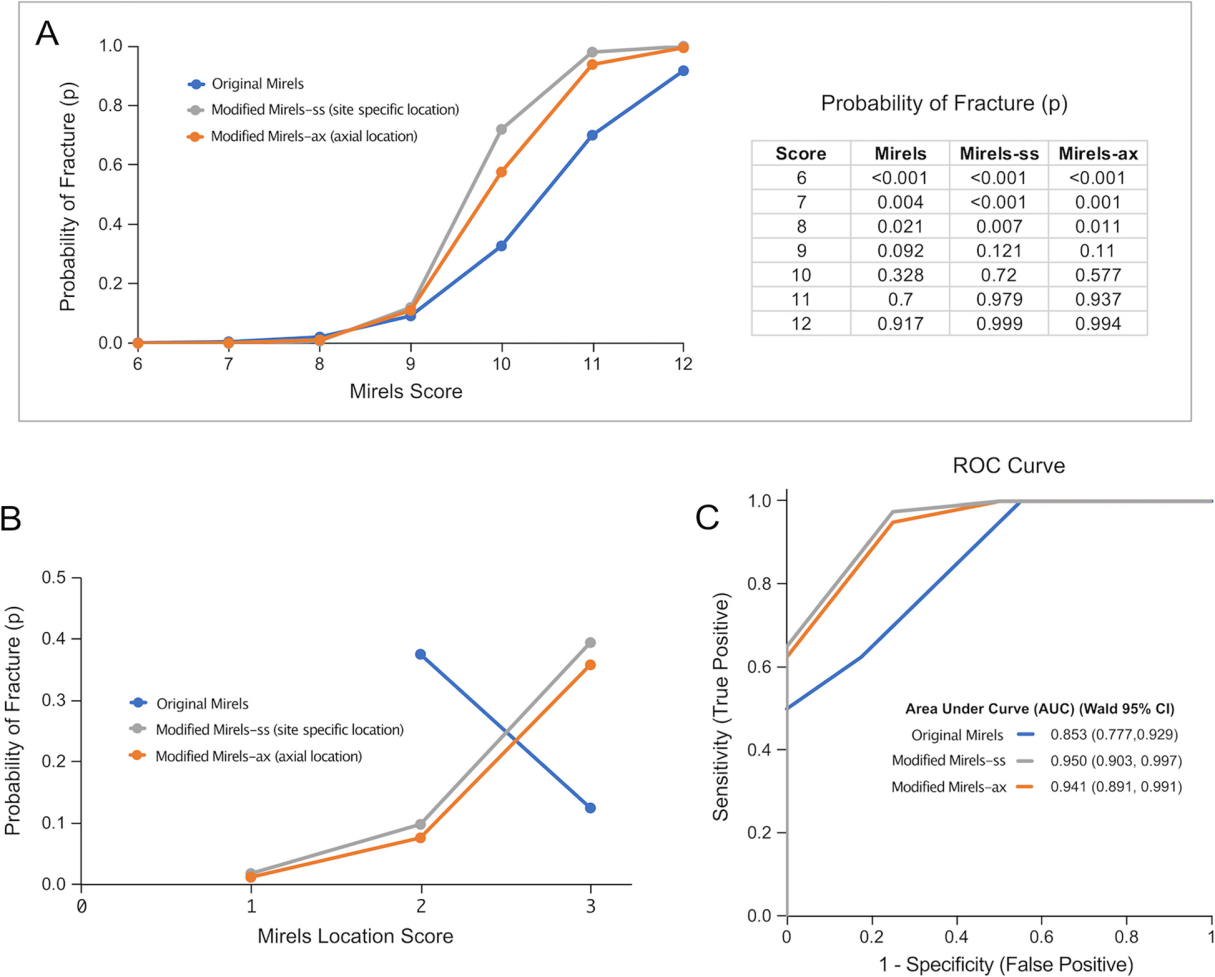
Logistic Regression Results. Legend: Probability of femur fracture for the 48 cases based on logistic regression for original Mirels and Modified Mirels scoring schemes (**A**). The effect likelihood ratio tests were significant (p < 0.0001) for original and both modified Mirels scoring. The probability of fracture for the Mirels location score (**B**). Receiver Operator Characteristic Curve (ROC) with area under curve (AUC) calculations (**C**) including 95% Wald-type confidence intervals for the AUC

The probability of fracture (Fig. 6A) calculated using logistic regression was similar for the original Mirels and modified Mirels scoring for composite scores of nine or less. For higher Mirels scores (> 9) the probability of fracture was greatest for the modified Mirels scoring with site specific location (modified Mirels-ss) followed by axial location (modified Mirels-ax). For a patient with a Mirels score of 10, the probability of fracture was 33% for original Mirels scoring, but increased to 56–72% for the modified Mirels scoring schemes. The confidence that a fracture would occur in this instance (Mirels score of 10) roughly doubles using the modified Mirels scoring when compared to original Mirels.

One would expect that a higher score would be associated with a higher probability of fracture, and this is found with the MMLS (Fig. 6B). But the reverse was found with the original Mirels location score (MLS); a case with a MLS of 2 had a higher probability of fracture compared to a case with a MLS of 3. Plotting results of sensitivity and specificity using Receiver Operator Characteristic (ROC) curves (Fig. 6C) showed that the modified Mirels scores improved predictive capabilities over Mirels scores and increased the Area Under Curve (AUC). However, it is difficult to interpret the magnitude of improvement in classification for modified Mirels scoring using AUC; net benefit analysis provides an alternative approach.

### Decision curve analysis and net benefit

**Fig. 7:**
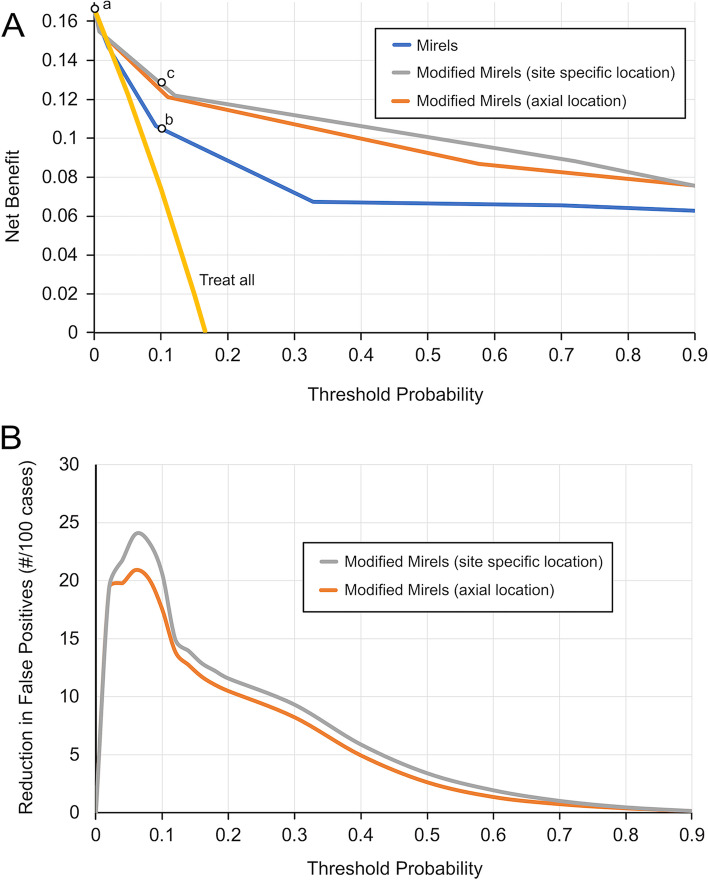
Decision curve analysis. Legend: The Net Benefit of using the original Mirels and modified Mirels scoring schemes as a function of threshold probability of fracture (**A**). A ‘treat all’ line is included representing the net benefit if all cases were considered to be at high risk of fracture. As an example, for p_t_ = 0.1 where a patient/surgeon is willing to accept a 10% chance of fracture, there was a net benefit to using the original Mirels scoring compared to treating all patients (point b). Further, there was an additional net benefit to using the modified Mirels scoring over original Mirels scoring at this threshold probability (point c). There is a net reduction in false positives using the modified Mirels scoring schemes over the original Mirels scoring (**B**) per 100 patients. For the example of p_t_ = 0.1, the modified scoring schemes reduced the number of false positives by 17% for the modified Mirels-ax and 20% for modified Mirels-ss

The net benefit of each of the Mirels scoring schemes (calculated using equation #1) represents the relationship between the acceptable threshold probability (x-axis) of allowing a fracture (without prophylactic intervention) and benefit of using the scoring scheme (y-axis) (Fig. 7A). A threshold probability (p_t_) of 0 represents the scenario of not allowing any fractures, and in this case, all femurs would be prophylactically stabilized. The ‘treat all’ line in the figure indicates this scenario and with p_t_ = 0, represents the prevalence of fractures (8 in 48 cases, NB = 0.17) in the sample population. There is no additional benefit of using Mirels scoring when p_t_ = 0 (point a in Fig. 7A). With increasing threshold probability beyond ~ 2%, both the original Mirels scoring scheme and modified scoring schemes have a greater net benefit when compared to treating all patients. In fact, the modified Mirels scoring provided an additional net benefit over Mirels scoring over a full range of threshold probabilities. The use of modified Mirels-ss provided a modest improvement over modified Mirels-ax in terms of net benefit.

There was a reduction in the number of false positives for the modified Mirels scoring schemes relative to the original Mirels scoring and this depended on the threshold probability (Fig. 7B). The reduction in false positives can also be illustrated by examining classification of impending fractures for the clinical series of patients (*n* = 48) with a Mirels score of 9 to 12. In this case, 100% of the true fractures were correctly identified for each of the scoring schemes, but the number of false positives was reduced from 55% for Mirels scoring to 35–38% modified Mirels scoring (Table 3).

**Table 3:** Outcomes of Mirels clinical classification

*Scoring System*	*Original Mirels*		*Modified Mirels-ss (site specific)*		*Modified Mirels-ax (axial)*	
*Clinical Outcome*	*Fx*	*No Fx*	*Fx*	*No Fx*	*Fx*	*No Fx*
Low Fx Risk (0–7)	0	7	0	17	0	15
Borderline (8)	0	11	0	9	0	10
Impending Fx (9–12)	8 (100% TP)	22 (55% FP)	8 (100% TP)	14 (35% FP)	8 (100% TP)	15 (38% FP)

Prediction of fracture (Fx) and no fracture (No Fx) cases using the three scoring schemes is shown for low, borderline, and impending fracture scores. Number of patients in each group are shown.

*TP* True positive, *FP* False positive

## Discussion

Numerous techniques have been proposed to predict risk of fracture in the setting of metastatic bone disease (MBD). A number of different approaches have been explored including lesion size [12, 30, 31], scoring systems that incorporate clinical and radiographic factors [12, 20], and structural analysis of bone strength using computed tomography (CT) data [1, 6, 16, 26, 28]. In the United States, the Mirels system is simple, widely taught, and based on readily available clinical and plain radiographic information alone [20]. CT-based techniques have superior specificity over Mirels but require expensive, time-consuming, complicated computational techniques not readily available to all and thus far not available in real time to inform timely clinical decision-making [1, 5, 6, 10, 22, 23, 26, 28]. A scoring system based on readily available information, whether plain radiographs or CT, without additional external analysis would enhance our ability to manage these patients, but only if it improves upon the original Mirels system. In this paper, a modified Mirels scoring system, informed by a detailed analysis of the influence of lesion location, improves the ability to predict proximal femur fractures in MBD. Two techniques, one requiring CT to identify axial and angular position of the lesion and the second only requiring axial position (which could be identified via plain radiographs), are shown to be nearly equal in their improvement over the original Mirels scoring.

### Limitations

This project had several important limitations. In this study, patients considered to be at low risk of fracture were followed without surgical intervention, while prophylactic stabilization was performed when deemed medically necessary. This resulted in a relatively small number of fracture cases; these were sometimes related to delays in operating on patients deemed as high risk (Table 2). The sample sizes available for this study were more than sufficient to confirm that either original Mirels score or modified Mirels score are effective predictors of fracture risk based on ROC Area Under Curve (AUC) metrics (MedCalc Software Ltd, Ostend, Belgium). To achieve a power of 0.8, 22 patients would be needed for the Modified Mirels Score (AUC = 0.95), and 44 patients would be needed for the Original Mirels Score (AUC = 0.853). Calculations into the sample size needed to establish statistically significant differences in AUC for the two scoring systems suggest that 70 patients (with equal sample sizes for fracture and no fracture groups) would provide power of 0.8 at the difference observed in this case. This could be achievable if x-ray evaluation were sufficient to assign a Modified Mirels score; it is likely more patients could be enrolled with x-ray imaging compared to the CT scan imaging used in this MSTS study database. Note that the AUC metric, while important, does not describe the shape of the ROC curve, and two scoring systems with the same AUC may perform better at certain combinations of true and false positives. The decision curves (Fig. 7) put this information into a format where comparisons can be made with any weighting of true positives and false positives. Here we show that the number of false positives can be reduced substantially with little loss of identifying true positives for clinically relevant fracture threshold probabilities.

A second limitation was that the computational models only included lytic lesions, and all lesions were the same idealized shape and relative size. However, lesion type and size were independently included in the Mirels scoring. Altering the relative shape or aspect ratio of the lesion would likely affect the fracture load of the femur, but these additional features would be difficult to document clinically and could cover a wide range of lesion conformations. The goal here was to use a parametric approach of adjusting lesion location, while keeping all other aspects of the lesion constant.

A third limitation was that the loading considered here simulated only stair ascent and was chosen because it represents a substantial biomechanical demand on the proximal femur. Clinically, patients with insufficiency fractures due to MBD may fracture during other activities such as rising from a chair, stair descent, or even higher loading situations such as stumbling or impact due to a fall. Patients with advanced cancer are known to have a higher risk of falling compared to an age-matched cancer free demographic [29]. Probabilistic methods could be used in the future to explore the uncertainties of loads on the femur based on patient population.

### Femoral strength reduction based on lesion location

The fracture strength of proximal femurs with simulated lesions was shown to be highly dependent on anatomic location of the lesions using computational finite element (FE) experiments. Femur strength with lesions ranged from 37 to 95% of the intact femur, suggesting that lesion location could be an important contributor to any scoring scheme. Femur strength was reduced the most for lateral lesions in the proximal diaphysis and subtrochanteric region, while posterior lesions in the intertrochanteric region and femoral neck had the least adverse effect. As a group, femurs with subtrochanteric and diaphyseal lesions lost the most strength, followed by intertrochanteric, with neck lesions having the least impact on femur strength. These findings are consistent with the reported strain distribution patterns of the proximal femur without lesions [15]. These findings conflict with the original Mirels location scoring approach where all pertrochanteric lesions were scored the weakest and diaphyseal lesions were scored at an intermediate level.

### Modified mirels location scoring

Informed by the FE findings, the location scoring of lesion location was modified using 1) a site specific modified location score (MMLS-ss) as determined by axial and angular locations on CT scan and 2) a simpler scheme condensed into four axial locations (MMLS-ax). Implementation into a modified Mirels score (MMS-ss or MMS-ax) only required modification of the location (site) component. Application of the two modified Mirels scoring schemes resulted in near equal improvement in fracture risk prediction for patients with proximal femoral MBD compared to original Mirels scoring using receiver operator characteristic (ROC) analysis. Note that CT data was used to identify lesion location, but the axial location of the lesions could likely be identified on planar radiographs. The use of MMS-ax scoring with radiographic images could have greater clinical utility compared to MMS-ss requiring CT scan data. It is also important to note that further FE analysis is not needed to assign the MMLS, one only needs to refer to the lookup table (Fig. 3).

### Decision curve analysis and net benefit

The final part of this study used decision curve analysis (DCA) to demonstrate the net benefit (NB) of using modified Mirels compared to the original Mirels scoring over a range of acceptable fracture threshold probabilities. Importantly, the two modified methods provided similar NB over the full range of threshold probabilities, supporting the concept that use of axial position of the lesion (as available via radiograph) may be sufficient in defining lesion location. Also note that the original Mirels scoring has a net benefit over treating all or no cases and thus has clinical value. For cases where Mirels guidelines (eg. prophylactic stabilization for 9 points or greater) are strictly applied, use of the modified scoring could reduce the number of false positives (patients predicted to fracture, but do not) by 17–20% without loss of missing true positive cases. This improvement is due to the high number of false positives with original Mirels scores of 9–12, indicating impending pathological fracture. For orthopedic surgeons caring for these patients, a primary goal is to prevent the well documented adverse effects of pathologic fractures by determining those patients at highest risk and prophylactically stabilizing those true positives. However, a balance is needed to avoid subjecting those patients without true impending fractures (false positives) to unnecessary surgery with its concomitant risks. DCA is a step towards understanding how to use that information in different clinical situations.

Although DCA is relatively new to orthopaedics [18, 33] and orthopaedic oncology [9, 25], it is being used increasingly across healthcare for diagnostic tests and treatment decisions [8]. In the setting of possible impending fracture in MBD, the decision curves inform the clinician as to which tool (Mirels vs modified Mirels) achieves the maximum net benefit to the patient at any given threshold probability over a broad spectrum. The results of this study found that modified Mirels scoring had a greater net benefit compared to Mirels scoring over the full range of threshold probabilities. In this case, one could conclude (assuming the same or similar results with an independent validation dataset) that modified Mirels should be used irrespective of what one would consider to be an appropriate threshold probability of fracture.

### What is an appropriate threshold probability?

Identifying an appropriate threshold probability requires consideration of the specifics of the clinical situation. In each instance, the surgeon and patient have to decide to what extent is it more important to prevent one fracture than to avoid unnecessary surgery. Some orthopedic oncologists would not want to perform more than 10 prophylactic surgical procedures to prevent one pathologic fracture, so their threshold would be 0.1 (10%). Note that the probability of fracture (Fig. 6) for an original Mirels score of 9 was 9%, which aligns closely with a threshold probability of 10%. Others might be willing to perform 20 procedures to prevent one fracture, which is equivalent to operating on a patient with a 5% risk of fracture. Considering the 30-day risk of major complications following prophylactic fixation in this setting is between 9.8 and 14%, performing surgery when the risk of fracturing is less than the risk of major complications may not be advisable [7, 19]. Regardless, this more aggressive approach might be warranted, based on a shared decision making, in a patient with a longer expected survival in order to avoid the even higher major complication risk (16–23%) and the poorer 1-yr survival after post-fracture fixation [7, 19]. By contrast, in some clinical scenarios where the risks of the prophylactic surgical procedure are increased or the patient’s prognosis is very poor, a less aggressive approach to proceeding to the operating room would be appropriate, perhaps a threshold probability of 0.2 (20%), where it is felt that preventing one fracture is only worth performing five prophylactic procedures. Avoiding one pathologic fracture in this scenario would be weighted as being only 5 times as important as preventing unnecessary surgery. Carefully incorporating relevant information on risk factors for complications, costs and expected survival would further inform selection of an appropriate threshold probability. For this study, the modified Mirels scoring provides the greatest reduction in false positives over this relevant 5 to 20% range as shown in Fig. 7B, indicating that modified Mirels scoring would be preferable over original Mirels scoring.

### Conclusion

In this study, we found that the strength of the proximal femur was highly dependent on the location of simulated metastatic lesion. The general pattern of greatest loss of strength for lesions in the subtrochanteric and proximal diaphysis was at odds with the traditional application of Mirels location score. There was an improvement in the ability to correctly predict fracture outcome for a series of clinical patients using the modified Mirels scoring system. Decision curve analysis showed that the modified Mirels scoring presented here reduces the number of false positives when compared to the original Mirels scoring (patients predicted to fracture, but do not) without loss of missing true positive cases over a full range of threshold probabilities of fracture. Independent validation of the modified Mirels scoring will be an important next step to verify utility of this approach.

## Supplementary Information

**Additional file 1.**

**Additional file 2.**

## Data Availability

Deidentified datasets used and analyzed during the current study are available as supplemental files with this submission.
