# Supplementary material for: Modification to Mirels scoring system location component improves fracture prediction for metastatic disease of the proximal femur
Source: BMC Musculoskelet Disord. 2023 Jan 24;24:65. doi: 10.1186/s12891-023-06182-7 (PMC9872372; doi:10.1186/s12891-023-06182-7)
Supplement: Supplementary file 1 — Additional file 1. [file 12891_2023_6182_MOESM1_ESM.docx]

**Additional File 1**

**Validating Finite Element Modeling Results for Simulated Lesions in the Proximal Femur**

**Goal**: To validate the utility of the non-linear finite element (FE) modeling approach to correctly quantify reduction in femur strength after introduction of simulated neck lesions. A second goal was to demonstrate that the FE modeling approach correctly identifies differences in reduction in femur strength for medial (inferior) and lateral (superior) neck lesions.

**Background and Rationale**

It is desirable to assess the validity of the FE modeling approach to gain confidence in the numerical results presented in this study. This is often accomplished by comparing FE simulations with clinical data (eg. do the models correctly predict femoral fracture risk for patients with metastatic lesions?) or carefully controlled biomechanical experiments performed in the laboratory. We previously reported on the use of the FE modeling approach to identify risk of fracture for patients with metastatic lesions using gait and stair ascent loading conditions [2]. In that study, the specificity of model predictions was improved for the finite element modeling approach when compared to the original Mirels scoring measures without loss of sensitivity. This suggests that the FE modeling approach that includes complex muscle and joint loading can improve femoral fracture prediction in the metastatic patient population.

Direct comparison of the current FE models with biomechanical tests using cadaver femurs is challenging for two reasons. First, it is difficult to apply stair ascent loading conditions to the greater trochanter for cases in which loading will progress to failure. Our previous experience [3] with stair ascent loading shows that it is possible to apply normal stair ascent loading, but increasing the loading to failure would often result in pullout of the simulated abductor mechanism. Second, it is difficult to experimentally create spherical lesions in the manner used in this study as the lesion shape would require precision expansion of the simulated defect moving inward from the periosteal bone surface. For this reason, we chose to validate the FE method using existing literature in which the reduction in strength of experimentally created lesions of the femoral neck using simple axial loading were determined [1].

**Approach**

In their experimental study, Benca et al [1] used 16 pairs of human femora with one femur remaining intact (control) and the other with a lesion reamed out in either the superolateral or inferomedial area of the femoral neck. The average age of the donors was 79.7 years with a range of 64 to 99 years old. There were 9 female and 7 male donors. To create the lesions, one-third of the cortex over the length of the superolateral or inferomedial neck was removed using an end mill. Axial compressive loads were applied to the femoral head to simulate weight on the femur during one-legged stance. Note that no abductor load was used. The results were reported in a paired sense (see Benca et al [1], Figure 8) with ultimate load for specimens with simulated lesions compared to contralateral intact femurs.

To validate the current FE method, superolateral and inferomedial neck lesions were placed in solid models of the 10 femurs of the No Lesion femur set (**Table 1**). The neck lesions used the same one-third of the cortex over the length of the neck as was used in the Benca experimental study. The FE meshes were created and material property assignment were made using the same approach as in the spherical lesion location study. Axial loading was applied to the femoral head in the same manner as the Benca experiment and the ultimate load to failure was determined for intact, superolateral, and inferomedial neck lesions.

**Results**

Benca et al reported that the inferomedial defects resulted in a greater loss of femur strength when compared to superolateral lesions as indicated by the open circles in Fig AF1. The results from the 10 FE models with simulated lesions showed a similar response (solid circles). The mean reduction in femur strength comparing experimental and FE results for inferomedial and superolateral lesions were not significantly different (p=0.21 and p=0.9, respectively) (Table AF1).

**Figure AF.1. Validation Analysis for Finite Element Model.**


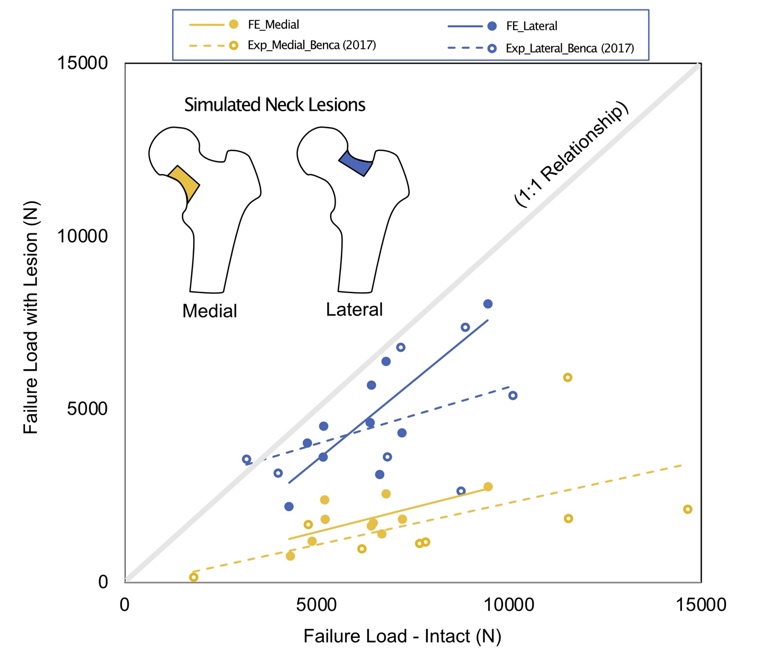


**Legend:** Experimental (open circle) data as reported in Figure 8 of the Benca dataset. Closed circle results of finite element (FE) modeling of simulated lesions in 10 independent proximal femurs.

**Table AF1**: Percentage reduction in femur strength for FE simulation and independent experimental data set.

| *Neck Lesion Location* | *Reduction in Femur Strength (%) - Finite Element Simulation* | *Reduction in Femur Strength (%) - Independent Experimental Data* | *t-test p-value* |
| --- | --- | --- | --- |
| Inferomedial | 71.2 _­_± 8.1 | 78.9 ± 14.4 | 0.21 |
| Superolateral | 26.2 ± 15.7 | 27.2 ± 28.2 | 0.90 |

Legend: The reduction in femur strength for lesions located in inferomedial and superolateral locations. Two sample t-tests were used to compared finite element and independent experimental data.

**Discussion**

The results of this validation study show that the FE modeling approach used here can capture the correct difference in strength pattern for superolateral and inferomedial lesions. Further, the differences in magnitudes of the strength reduction due to the lesions comparing FE models and experiments was small (~7% and 1% for inferomedial and superolateral lesions, respectively). For femurs with spherical lesions used in the main study, the loss of strength ranged from 7 to 62% (based on Table 3 data) depending on location. This range is much larger than the errors found between experimental data and FE modeling reported in this validation study. This suggests that the FE modeling approach used here is sufficient to assess the spatial effect of spherical lesion location on femur strength.

**REFERENCES**

1. Benca E, Reisinger A, Patsch JM, Hirtler L, Synek A, Stenicka S, Windhager R, Mayr W, Pahr DH. Effect of simulated metastatic lesions on the biomechanical behavior of the proximal femur. *J Orthop Res.* 2017;35:2407-2414.

2. Goodheart JR, Cleary RJ, Damron TA, Mann KA. Simulating activities of daily living with finite element analysis improves fracture prediction for patients with metastatic femoral lesions. *J Orthop Res.* 2015;33:1226-1234.

3. Race A, Miller MA, Clarke MT, Mann KA, Higham PA. The effect of low-viscosity cement on mantle morphology and femoral stem micromotion: A cadaver model with simulated blood flow. *Acta Orthop.* 2006;77:6007-6616.
