# Supplementary material for: Modification to Mirels scoring system location component improves fracture prediction for metastatic disease of the proximal femur
Source: BMC Musculoskelet Disord. 2023 Jan 24;24:65. doi: 10.1186/s12891-023-06182-7 (PMC9872372; doi:10.1186/s12891-023-06182-7)
Supplement: Supplementary file 2 — Additional file 2. [file 12891_2023_6182_MOESM2_ESM.docx]

Title: Modified Mirels Scoring Components.


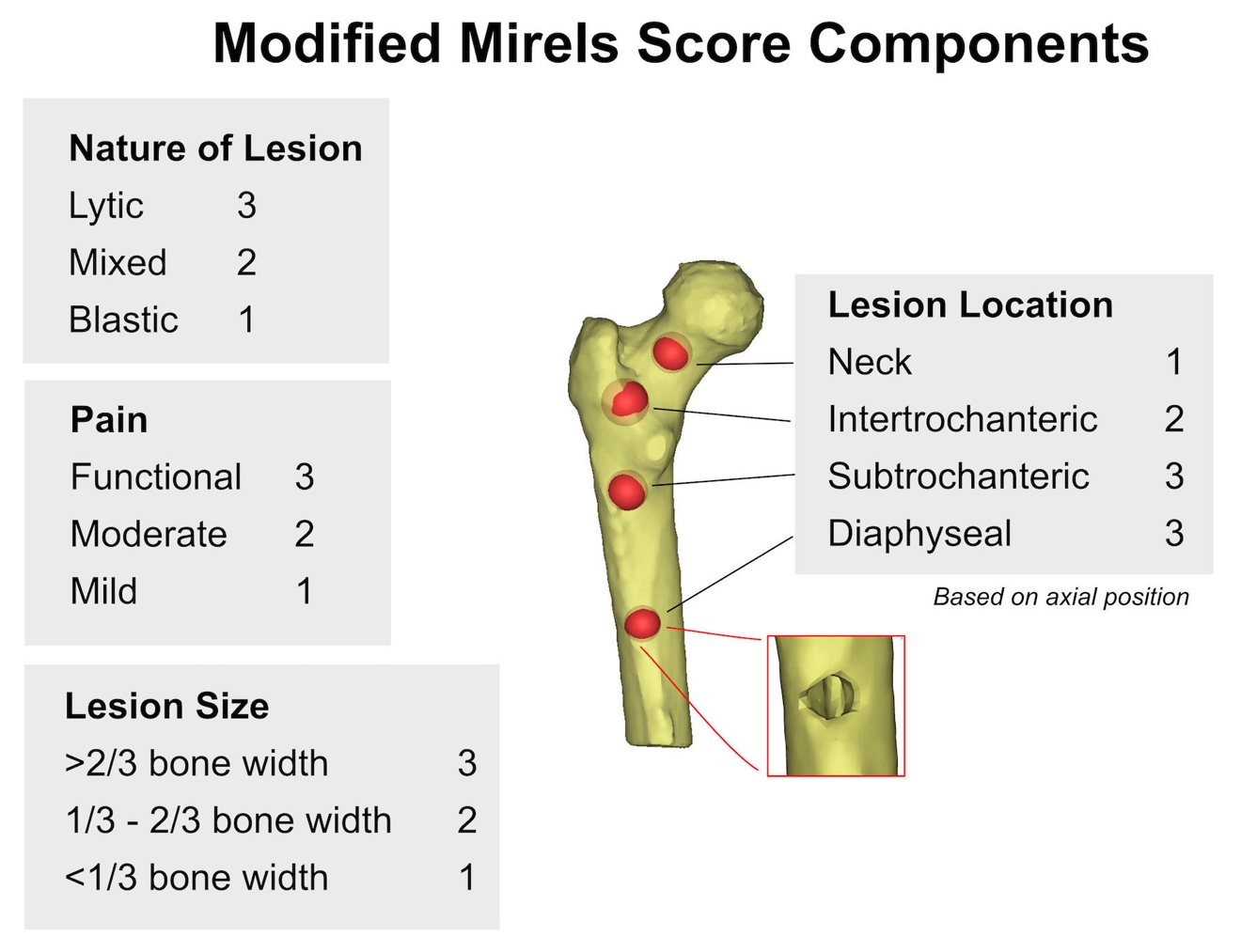


Legend: Lesion location scoring is based on axial position.
